# Melatonin Improves Cotton Salt Tolerance by Regulating ROS Scavenging System and Ca^2 +^ Signal Transduction

**DOI:** 10.3389/fpls.2021.693690

**Published:** 2021-06-28

**Authors:** Yuexin Zhang, Yapeng Fan, Cun Rui, Hong Zhang, Nan Xu, Maohua Dai, Xiugui Chen, Xuke Lu, Delong Wang, Junjuan Wang, Jing Wang, Qinqin Wang, Shuai Wang, Chao Chen, Lixue Guo, Lanjie Zhao, Wuwei Ye

**Affiliations:** State Key Laboratory of Cotton Biology/Institute of Cotton Research of Chinese Academy of Agricultural Sciences/Research Base, Zhengzhou University/Key Laboratory for Cotton Genetic Improvement, MOA, Anyang, China

**Keywords:** cotton, melatonin, salt stress, Ca^2+^, ROS

## Abstract

As one of the cash crops, cotton is facing the threat of abiotic stress during its growth and development. It has been reported that melatonin is involved in plant defense against salt stress, but whether melatonin can improve cotton salt tolerance and its molecular mechanism remain unclear. We investigated the role of melatonin in cotton salt tolerance by silencing melatonin synthesis gene and exogenous melatonin application in upland cotton. In this study, applicating of melatonin can improve salt tolerance of cotton seedlings. The content of endogenous melatonin was different in cotton varieties with different salt tolerance. The inhibition of melatonin biosynthesis related genes and endogenous melatonin content in cotton resulted in the decrease of antioxidant enzyme activity, Ca^2+^ content and salt tolerance of cotton. To explore the protective mechanism of exogenous melatonin against salt stress by RNA-seq analysis. Melatonin played an important role in the resistance of cotton to salt stress, improved the salt tolerance of cotton by regulating antioxidant enzymes, transcription factors, plant hormones, signal molecules and Ca^2+^ signal transduction. This study proposed a regulatory network for melatonin to regulate cotton’s response to salt stress, which provided a theoretical basis for improving cotton’s salt tolerance.

## Introduction

Cotton is an industrial and cash crop with important cash value (Sunilkumar et al., 2006). During the growth and development of cotton, it is often subjected to some abiotic stresses, such as drought, salinity, high temperature, and low temperature. Soil salinization is one of the biggest challenges facing world agriculture (Zhu, 2001), because salinization hinders the growth and development of plants and reduces crop yields (Shahzad et al., 2021). The saline soil contains excessive neutral salt, mainly NaCl and Na_2_SO_4_, which cause salt stress. The harmful effects of NaCl on plants are due to the accumulation of sodium in the soil, which reduces water availability and the toxic effects of sodium and chloride ions on plants (Van-Zelm et al., 2020). Plants use a variety of biochemical and molecular responses to cope with stress, including selective formation or elimination of salt ions, control of root absorption of ions and transport to leaves, synthesis of compatible osmotic agents, stimulation of hormones, regulation of gene expression, etc. (Parida and Das, 2005). More than 800 million hectares of land throughout the world are salt affected. This amount accounts for more than 6% of the world’s total land area (Munns and Tester, 2008). Therefore, the yield of salinized farmland is increased by improving the salt-tolerance of cotton, studying the salt tolerance of cotton has become more and more important.

Many plant growth regulators have been used to improve the salt tolerance of crops to achieve agricultural sustainability, such as SA (salicylic acid) (Bastam et al., 2013). Melatonin is a growth regulator and stress regulator, which can improve plant performance and yield (Arnao and Hernández-Ruiz, 2014). Both exogenous application of melatonin and genetic transformation of melatonin synthesis genes to regulate endogenous melatonin concentration can alleviate the effects of biotic and abiotic stress. Melatonin has been reported to improve plant tolerance to salt stress. Exogenous melatonin alleviates reactive oxygen species (ROS) accumulation and protects photosynthetic activity in Maize Seedlings under salt stress by activating antioxidant enzymes (Chen et al., 2018). Melatonin improved tolerance to salt stress by promoting growth, root yield and sugar content, chlorophyll synthesis, photosynthetic system II (PS II) activity, and changed in gas exchange parameters in sugar beet seedlings (Zhang et al., 2021). Melatonin enhances Phaseolus vulgaris salt tolerance by enhancing ROS metabolism, expression of antioxidant defense related genes, and photosynthetic capacity (ElSayed et al., 2021). Melatonin affects rice photosynthesis under salt stress by increasing total antioxidant capacity, promoting the xanthophyll cycle, increasing xanthophyll pool size to dissipate excess light energy, increasing key photosynthetic enzyme activities, and maintaining a low ROS state (Yan et al., 2021). Interaction of Ca/CaM and melatonin is involved in overcoming salt-induced ionic, osmotic, and oxidative damages and Ca and melatonin may act as long-distance signals for inducing systemic salt tolerance in *Dracocephalum kotschyi* (Vafadar et al., 2020). The increase in melatonin caused by the high expression of *MzASMT9* leads to *Arabidopsis* strains with higher salt tolerance than wild-type plants, which can be proved by reducing ROS, reducing lipid peroxidation and enhancing photosynthesis (Zheng et al., 2017).

Melatonin (N-acetyl-5-methoxytryptamine) is a metabolite derived from tryptophan, and it is widely distributed in primitive photosynthetic bacteria and higher plants, including algae and fungi (Kanwar et al., 2018). In 1995, melatonin was first discovered in plants (Dubbels et al., 1995; Hattori et al., 1995), since then, the research on plant “melatonin” has started. Melatonin is related to many physiological functions of plants, including seed germination, growth, rooting, photosynthesis and stress resistance. It is considered to be a multi-regulatory molecule and may play a role in plant master regulation (Arnao and Hernandez-Ruiz, 2019). In plants, melatonin is synthesized from tryptophan through four successive enzyme reactions (Kang et al., 2011), including tryptophan decarboxylase (TDC) (Luca and Brisson, 1989), tryptamine 5-hydroxylase (T5H) (Fujiwara et al., 2010), serotonin N-acetyltransferase (SNAT) (Lee et al., 2014), N-acetylserotonin methyltransferase (ASMT) (Kang et al., 2011). It is reported that caffeic acid O-methyltransferase (COMT) also has ASMT enzyme activity in Arabidopsis (Byeon et al., 2014). Although there has been a lot of research on melatonin in abiotic stress, the molecular mechanism of melatonin in regulating cotton response to NaCl stress is still unclear.

In this study, 20 μM melatonin was used to pre-treat cotton to explore the molecular mechanism of melatonin improving salt tolerance of cotton. We analyzed the differentially expressed genes (DEGs) induced by melatonin in cotton involved in salt stress by RNA-seq technology, and found that DEGs are involved in phosphoinositide metabolism and signal transduction, hormone synthesis and signal transduction, and redox reactions, some transcription factors were only induced by melatonin. Inhibition of the expression of the melatonin synthesis gene *GhCOMT* by virus induced gene silencing technology (VIGS) suppressed the endogenous melatonin content in cotton, resulting in the silenced plants being more sensitive to salt stress, and exogenous supplementation of melatonin to the silenced plants alleviated the salt sensitivity of the silenced plants. Meanwhile, there were differences in melatonin content between two cotton materials with different salt tolerance (Zhong9807 and GK50), and Zhong9807 melatonin contents were higher in cotton materials with high salt tolerance than in GK50. This study aims to use melatonin to improve the salt tolerance of cotton, to explore the mechanism of melatonin in regulating cotton’s response to salt stress, to discover salt tolerance genes regulated by melatonin, and to provide a new basis for improving the salt tolerance of cotton.

## Materials and Methods

### Plant Materials and Growth Conditions

Taking upland cotton cultivar Zhong9807 and GK50 as experimental materials, Zhong9807 has higher salt tolerance than GK50 (Wang et al., 2016). The cotton seeds were sown on a 1:1.5 medium substrate of sand and vermiculite, and grown in an indoor incubator at 25°C for 16 h during the day and 8 h at night. In the treatment of cotton seedlings, the washed cotton seedlings were placed in 300 ml volumetric Erlenmeyer flasks with 10 seedlings in each Erlenmeyer, and the Erlenmeyer flasks were placed in a room incubator at 25°C for 16 h during the day and 8 h during the night.

### Pre-treatment of Cotton With Exogenous Melatonin

To explore the suitable concentration of melatonin for improving salt tolerance in cotton, we washed three leaf stage cotton seedlings, moved to Erlenmeyer flasks containing 300 ml capacity of distilled water, and 10 seedlings in each Erlenmeyer flask. Cotton seedlings were treated with different concentrations of melatonin (0, 20, 50, 100, and 200 μM) and sprayed once a day for three consecutive days in a constant temperature incubator at 25°C for 16 and 8 h during the day/night. The melatonin pre-treated cotton seedlings were retransplanted to 100 mM/L NaCl solution Erlenmeyer flasks, 0 μM concentration of melatonin treated cotton seedlings as the blank control (CK), water-treated wild-type seedlings were used as reference for normal growth (CK0) and phenotypic changes were observed.

### cDNA Library Preparation and Transcriptome Sequencing

The true leaf samples of cotton seedlings pre-treated with 0 μM or 20 μM melatonin were collected for transcriptomic sequencing 12 h after salt treatment. Cotton seedlings pre-treated with 0 μM melatonin were used as controls and three biological replicates were set. Total RNA was extracted using the RNAprep Pure Plant Kit (Tiangen, Beijing, China) according to the manufacturer’s instructions. RNA degradation and contamination were monitored on 1% agarose gels. RNA purity was checked using the NanoPhotometer^®^ spectrophotometer (IMPLEN, Westlake Village, CA, United States). RNA concentration was measured using Qubit^®^ RNA Assay Kit in Qubit^®^ 2.0 Flurometer (Life Technologies, Carlsbad, CA, United States). RNA integrity was assessed by using the RNA Nano 6000 Assay Kit of the Agilent Bioanalyzer 2100 system (Agilent Technologies, Santa Clara, CA, United States). A total amount of 1 μg RNA per sample was used as input material for the RNA sample preparations. Sequencing libraries were generated using NEBNext^®^ Ultra^TM^ RNA Library Prep Kit for Illumina^®^ (NEB, United States) following manufacturer’s recommendations and index codes were added to attribute sequences to each sample. The clustering of the index-coded samples was performed on a cBot Cluster Generation System using TruSeq PE Cluster Kit v4-cBot-HS (Illumia) according to the manufacturer’s instructions. After cluster generation, the library preparations were sequenced on an Illumina Hiseq 2500 platform and paired-end reads were generated.

### RNA-Seq Data Analysis

Raw data (raw reads) of FASTQ format were firstly processed through in-house perl scripts. In this step, clean data (clean reads) were obtained by removing reads containing adapter, reads containing ploy-N and low-quality reads from raw data. At the same time, Q20, Q30, GC content and sequence duplication level of the clean data were calculated. All the downstream analyses were based on clean data with high quality.

The clean reads were mapped to the reference genome sequence, and reads that were perfectly matched or contained one mismatch were further analyzed and annotated on the basis of the reference genome. We used HISAT2 tools to map the reads to the reference genome. Gene expression levels were estimated as fragments per kilobase of transcript per million fragments mapped (FPKM).

Differential expression analysis of two conditions/groups was performed using the DESeq R package (1.10.1). DESeq provide statistical routines for determining differential expression in digital gene expression data using a model based on the negative binomial distribution. The resulting *P* values were adjusted using the Benjamini and Hochberg’s approach for controlling the false discovery rate. Genes with an adjusted *P*-value < 0.05 found by DESeq were assigned as differentially expressed. Consequently, DEGs were obtained of three biological conditions. Fold Change ≥ 2 and FDR < 0.01 were taken as the thresholds for determining whether a gene had differential expression.

Gene Ontology (GO) enrichment analysis of the DEGs was implemented by the GOseq R packages based Wallenius non-central hyper-geometric distribution (Young et al., 2010), which can adjust for gene length bias in DEGs. KEGG (Kanehisa et al., 2007) is a database resource for understanding high-level functions and utilities of the biological system, such as the cell, the organism and the ecosystem, from molecular-level information, especially large-scale molecular datasets generated by genome sequencing and other high-throughput experimental technologies^1^. We used KOBAS (Mao et al., 2005) software to test the statistical enrichment of differential expression genes in KEGG pathways.

### Real-Time PCR

Randomly selected 15 different genes and used the same sample to perform qRT-PCR to verify the RNA-seq data. The total RNA was extracted with EASYspin Plus plant RNA rapid isolation kit (Aidlab Co., Ltd., Beijing, China). The pure RNA was reverse-transcribed using Transcript United States II one-step gDNA removal and cDNA synthesis supermix (TransGen Biotech Co., Ltd., Beijing, China) according to the manufacturer’s instructions. Primer Premier 5 software was used to design gene-specific primers, details of primers are shown in Supplementary Table 2. qRT-PCR assays were performed on the Bio-Rad 7500 fast fluorescence quantitative PCR platform with TransStart^®^ top green qPCR supermix (TransGen Biotech Co., Ltd., Beijing, China) in accordance with the manufacturer’s protocol, three biological replicates, the 2^–Δ^ ^Δ^ ^*Ct*^ method is used to measure the relative expression level of genes (Livak and Schmittgen, 2002). Internal control is *GhUBQ7* (Tan et al., 2013), it is stably expressed in cotton plants and is not affected by treatment and genotype. Perform correlation analysis between qRT-PCR and RNA-seq.

### Effect of Salt Stress on Melatonin in Cotton

To explore the change of melatonin content in cotton under salt stress, we washed Zhong9807 cotton seedlings of three leaf stage size, transplanted them into a 300 ml volumetric Erlenmeyer flask containing 100 mM/L NaCl solution, and at 0 and 12 h of salt treatment, the true leaves were taken as samples for melatonin determination in three biological replicates.

### Determination of Melatonin Content in Different Cotton Materials

In order to explore whether the endogenous melatonin level will affect the salt tolerance of cotton varieties, we selected medium Zhong9087 and GK50 cotton seedlings of the three-leaf stage size, and took true leaves as samples for determining the melatonin content, three biological replicates for each sample.

### Endogenous Melatonin Content Detection

Samples needed to measure endogenous melatonin levels were taken, and used the Plant Melatonin (MT) ELISA Kit (Ziker, ZK-P7490, Shenzhen, China) to measure the endogenous melatonin content. The kit uses double-antibody one-step sandwich enzyme-linked immunosorbent assay (ELISA). Add 0.1 g of the sample to an appropriate amount of physiological saline, mash it, centrifuge at 3000 rpm for 10 min, and take the supernatant. The assay was performed according to the instructions of the Plant Melatonin (MT) ELISA Kit, with three biological replicates for each sample.

### Detecting of Antioxidant Enzyme Activity

Samples needed to determine the peroxidase (POD) activity and superoxide dismutase (SOD) activity were taken, and used the POD activity detection kit (SinobestBio, YX-W-A502, Shanghai, China) and the SOD activity detection kit (SinobestBio, YX-W-A500-WST-8, Shanghai, China) to determine the antioxidant enzyme activity, respectively. Weighed about 0.1 g of tissue and added 1 mL of extract for ice bath homogenization; centrifuged at 8000 × *g* at 4°C for 10 min, supernatant was placed on ice and measured according to the instructions. Three biological replicates were performed for each sample.

### Detecting of Ca^2+^ Content

The determination of Ca^2+^ content refers to EDTA titration method in GB 5009.92–2016 “Determination of Calcium in Food of National Standard for Food Safety.” Samples that needed to be assayed for Ca^2+^ content were cleaned by ddH_2_O, placed in an oven, oven dried at 110°C for 10 min, 80°C until constant weight, accurately weigh 0.2 g of sample into a graduated digestion tube, add 10 ml 10% nitric acid, and digest in an adjustable electric furnace (reference conditions: 120°C/0.5 h to 120°C/1 h, increase to 180°C/2 h to 180°C/4 h, and increase to 200–220°C). The digestion solution appeared colorless and transparent or slightly yellow. Constant volume to 25 ml with water after cooling, then dilute as needed for the actual assay, and add a volume of lanthanum solution (20 μg/L) to the dilution to a final concentration of 1 μg/L and mix for further use, this being the sample to be tested. Pipette 1 ml of the sample to be tested and a blank into a test tube and add one drop of sodium sulfide solution (10 g/L), 0.1 ml of sodium citrate solution (0.05 mol/L), 1.5 ml of potassium hydroxide solution (1.25 mol/L), and three drops of calcium red indicator. Titrate immediately in a 10 fold dilution of EDTA solution until the indicator changes blue from purple red and record the volume of EDTA solution consumed with a 10 fold dilution.

### VIGS Technique Silenced the Melatonin Synthesis Gene

In order to investigate whether the content of endogenous melatonin in cotton has an effect on the salt tolerance of cotton, the melatonin synthesis gene was silenced by VIGS. We used *Arabidopsis AtCOMT* (AT5G54160) (Byeon et al., 2014) as the query sequence and BLAST to the cotton genome to obtain the putative *GhCOMT* (Gh_D12G2680) in cotton. Vector was a pYL156 vector maintained in our laboratory, and BamHI and SacI restriction enzymes were selected for double digestion, Use the online tool SGN–VIGS^2^ design length in 300 bp silence about the size of the fragment. The in-Fusion primers for *GhCOMT* were designed manually, and the primers for the *GhCOMT* silencing fragment were as follows: forward primer, 5′-AGAAGGCCTCCATGGGGATCC ATGGGTTCAACCGGTGAAACCCAAAT-3′; reverse primer, 5′-TGCCCGGGCCTCGAGACGCGTGAGCTCGCCATCAGGA AGAGTGCGCA-3′. Using cotton leaf cDNA as template, the silenced fragment was amplified, and the VIGS expression vector *pYL156:GhCOMT* was constructed by in-Fusion ligation technique. The constructed expression vectors were transformed into *Escherichia coli*, and after correct sequencing, they were transformed into Agrobacterium using the freeze-thaw method. The virus mediated gene silencing (VIGS) system consisted of a recombinant vector, the negative control pYL156, the positive control *pYL156:PDS*, and the helper vector pYL192. Silencing of the phytoene dehydrogenase gene (*PDS*) resulted in white leaves, so the whole system was judged to be correct by observing whether the VIGS plants with *pYL156:PDS* had true leaves turned white. After the cotyledon of cotton seedling was flattened, it was prepared for infection. The night before infection, sufficient water was poured, and the Agrobacterium solution was handled well. After the injection was completed, cotton seedlings were incubated normally after they were protected from light for 24 h. Cotton was treated with 100 mm NaCl when grown to the three leaf stage.

### Statistical Analysis

The GraphPad Prism 8.0 software was employed to analysis (ANOVA) the results. Duncan’s Multiple Range Test was used to compare the least significant difference of means (*P* < 0.05).

## Results

### Exogenous Application of Melatonin Improves Cotton Salt Tolerance

**FIGURE 1:**
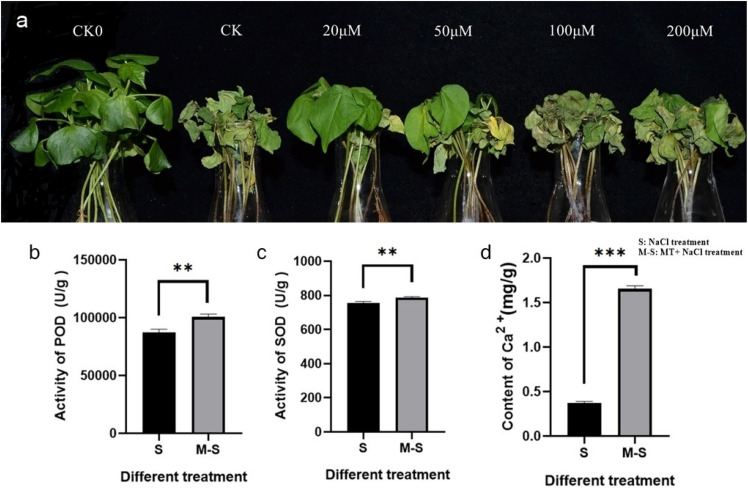
Melatonin enhances salt tolerance in cotton. **(a)** Wilting symptoms 4 days after cotton salt pre-treatment with different concentrations of melatonin (0, 20, 50, 100, and 200** μ** M); **(b)** Melatonin enhances the activity of POD; **(c)** Melatonin enhances the activity of SOD; **(d)** Melatonin enhances the content of Ca^2^**^+^.** **p < 0.01 and ***p < 0.001.

Cotton seedlings at three leaf stages were pre-treated with different concentrations of melatonin (0, 20, 50, 100, and 200 μM), and then treated with 100 mM NaCl solution, 0 μM concentration of melatonin treated cotton seedlings as the blank control (CK), water-treated wild-type seedlings were used as reference for normal growth. Different responses to salt stress were observed in melatonin-pre-treated and control seedlings (Figure 1a). At 12 h salt treatment, the cotyledons of the control seedlings lost luster and wilting more severely than the pre-treated seedlings. With the increase of salt stress time, the true leaves of control seedlings wilted at first, and the wilting degree was more serious than that of pre-treated seedlings. At 4 days of salt stress treatment, almost all the control seedlings died, but the seedlings treated with 20 or 50 μM melatonin did not die completely. Although all the cotyledons of the seedlings treated with 20 μM melatonin wilted, the true leaves only partially wilted and did not die. Therefore, Therefore, 20 μM melatonin was preliminarily selected as the treatment concentration based on phenotypic changes.

In order to test whether 20 μM melatonin could improve salt tolerance of cotton, antioxidant enzyme activity and Ca^2+^ content were measured. The antioxidant enzyme activity and Ca^2+^ content of seedlings treated with 0, 20 μM melatonin were determined after salt stress. The POD activity (Figure 1b), SOD activity (Figure 1c), and Ca^2+^ content (Figure 1d) were significantly different among different treatments. Under salt stress, the POD activity, SOD activity, and Ca^2+^ content of plants treated with exogenous melatonin increased significantly.

### Analysis of the Response of DEGs to Salt Stress Under Melatonin Pre-treatment

In order to explore how exogenous melatonin regulates cotton’s response to salt stress and thus improves salt tolerance, we compared the RNA-seq data of CK VS S and S VS M-S. We used the salt stress treatment for 12 h as the sampling time. The samples included the control group (CK) and the treatment group (S, M-S), and three biological replicates. Transcriptome analysis of nine samples, a total of 58.98 Gb Clean Data was obtained, and the Clean Data of each sample reached 5.83 Gb, and the Q30 base percentage was 93.03% and above (Supplementary Table 1). Clean Reads of each sample were sequentially aligned with the specified reference genome, the comparison efficiency varies from 96.17 to 97.26%. Based on the results of the comparison, we performed alternative splicing prediction analysis, gene structure optimization analysis, and discovery of new genes. 7608 new genes were discovered, of which 6588 were functionally annotated.

**FIGURE 2:**
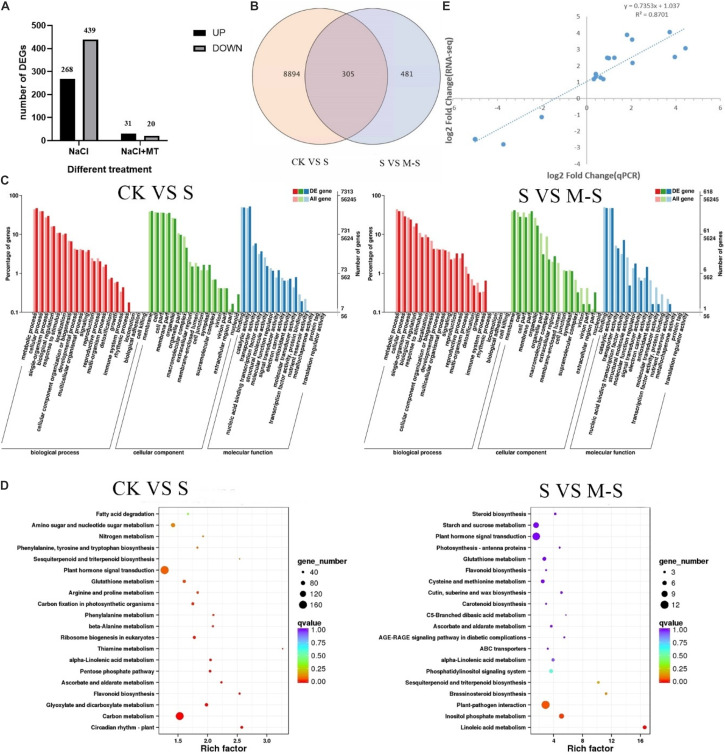
Results of RNA-seq data analysis. **(A)** Number of different gene expression (DEGs) in CK VS S and S VS M-S. **(B)** Venn diagram of DEGs. **(C)** Summary of Gene Ontology (GO) categories of the DEGs. **(D)** Summary of KEGG categories of the DEGs. **(E)** Relationship between RNA-seq and quantitative real-time PCR (Q-PCR) expression data (log_2_ fold change) (*R*^2^ = 0.8701) Note: CK: Blank Control; S: Salt stress treatment; M-S: Melatonin and salt stress treatment.

In the process of DEGs detection, Fold Change ≥ 2 and FDR < 0.01 are used as the screening criteria to obtain the differential gene expression (DEGs) between the two treatments (CK VS S and S VS M-S) (Figure 2A). In the CK VS S library, we detected 9199 DEGs that responded to salt stress (compared to CK, 3837 genes were up-regulated and 5362 genes were down-regulated). In the SV S M-S library, a total of 786 DEGs were detected (relative to S, 356 genes were up-regulated and 430 genes were down-regulated). Figure 2B showed the differences in DEGs among the three treatments. We found that 305 genes were differentially expressed jointly between S and M-S, 8894 genes were specifically expressed in the treatment group S, and 481 genes were specifically expressed in the treatment group M-S. These DEGs are related to the relief of salt stress by melatonin. These genes that respond to exogenous melatonin were considered to be important candidate genes for further research.

According to the classification method of GO, the functions of each classification system are classified (Young et al., 2010). A total of 7,313 DEGs between CK and S were annotated by GO, most of which were rich in functional categories such as metabolic process, cell process, membrane component, binding and catalytic activity. Between S and M-S, 618 DEGs were co-enriched by GO annotation (Figure 2C), which were mainly divided into metabolic process, cell process, membrane part, cell part, binding activity and catalytic activity, etc.

Differentially expressed genes were performed KEGG enrichment analysis to determine the main pathways for the enrichment of melatonin-induced cotton leaves under salt stress (Figure 2D). Between CK and S, 1872 DEGs enriched to 121 KEGG pathways, mainly enriched in the following pathways: there were 163 genes in plant hormone signal transduction (ko04075) (38 genes were up-regulated, 125 genes were down-regulated); 156 genes were enriched in carbon metabolism (ko01200), and there were 120 genes in starch and sucrose metabolism (ko00500). Between S and M-S, there were 12 genes (11 genes down-regulated, 1 gene up-regulated) in plant-pathogen interaction (ko04626), and 11 genes (8 genes up-regulated, 3 genes down-regulated) were enriched to plant hormone signal transduction (ko04075), there were seven genes (six up-regulated and one down-regulated) in inositol phosphate metabolism (Ko00562). A total of five genes (three up-regulated and one down-regulated) were enriched to phosphatidylinositol signaling system (KO04070) and five genes up-regulated in Glutathione metabolism (Ko00480). These pathways were main enrichment pathways.

To verify the transcriptome data, we used qRT-PCR to explore the expression of 15 different genes randomly selected. Perform correlation analysis on the two sets of data, qRT-PCR and RNA-seq were used to compare the gene folding changes (FC) between the two treatment groups. As shown in the Figure 2E, the qRT-PCR data were consistent with the RNA-seq data, and the significant positive correlation (*R*^2^ = 0.8701) supports the reliability of the RNA-seq data.

### Melatonin Regulates the Expression of Redox-Related Genes to Relieve Salt Stress

In order to explore the oxidoreductase related genes involved in salt stress regulation by melatonin, we found that 707 genes were enriched in oxidoreductase activity (GO:0016491), 439 genes were down-regulated, and 268 genes were up-regulated. Between S and M-S, 51 genes were found to have the molecular function of oxidoreductase activity (GO:0016491), of which 20 genes were down-regulated and 31 genes were up-regulated. In total, 50 of these genes were assigned to the oxidation-reduction process (GO:0055114), and they were differentially expressed in each treatment. Among them, 18 genes were down-regulated under salt stress, but were up-regulated by melatonin under salt stress; six genes were up-regulated under salt stress, but were induced down-regulated by melatonin under salt stress (Supplementary Table 3). The specific expression of seven genes was induced by melatonin under salt stress. We found that 20 redox-related genes were not expressed under salt treatment alone, but were regulated by melatonin under salt stress and their expression is up-regulated. These results indicated that melatonin can improve cotton salt tolerance by regulating some redox-related genes.

### Melatonin Regulates Transcription Factors in Response to Salt Stress

**FIGURE 3:**
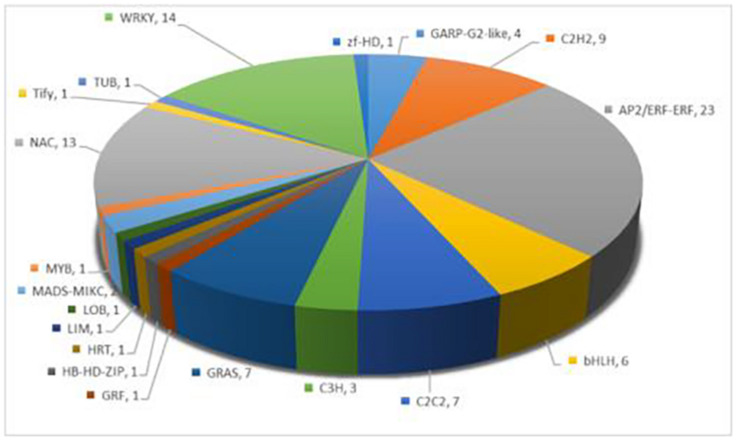
Annotation of specific expression of transcription factors induced by melatonin under salt stress.

Transcription factors play a vital role in regulating plant stress tolerance (Gruber et al., 2009). In order to discover the transcription factors induced by melatonin under salt stress, a total of 98 transcription factors were differentially expressed between S and M-S, including AP2/ERF-ERF, WRKY, NAC, and C2H2 and other transcription factor family members (Figure 3). Interestingly, 60 transcription factors were not differentially expressed between S and CK, but were differentially expressed between S and MS. There were 23 transcription factors that were up-regulated by melatonin under salt stress, such as AP2/ERF-ERF, C2H2, bHLH, and WRKY. These transcription factors were not induced by salt stress, but were regulated by melatonin under salt stress, and participate in cotton’s resistance to salt stress.

### Melatonin Co-ordinates Other Plant Hormones in Cotton’s Resistance to Salt Stress

**FIGURE 4:**
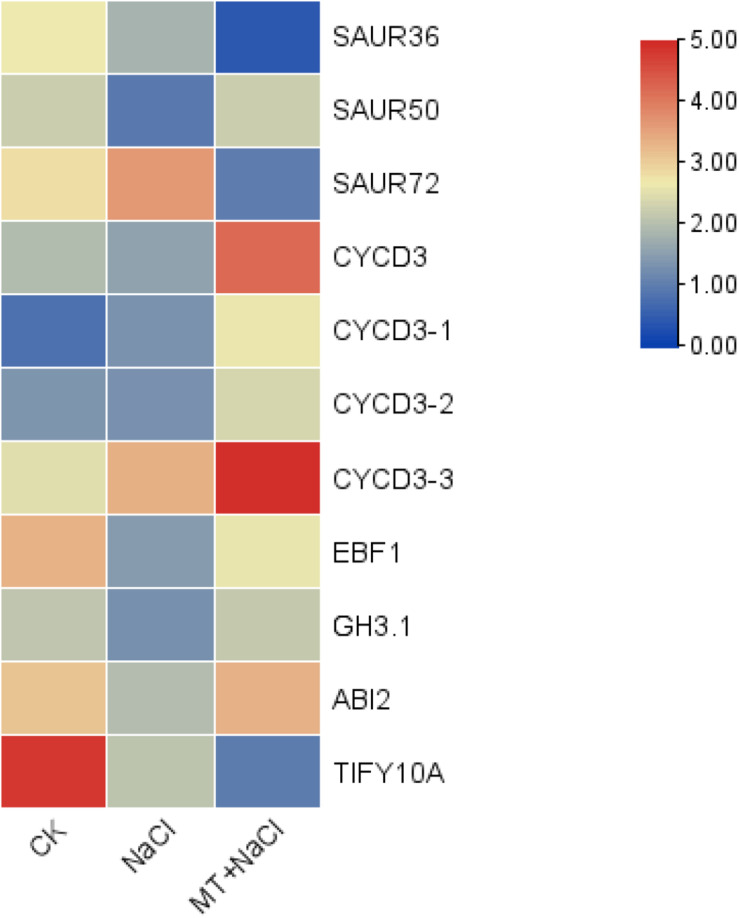
Effect of melatonin on gene expression related to cotton hormone signal transduction.

As we all know, plant hormones play a very important role in plant growth and stress response. The idea of melatonin as a plant hormone is gradually accepted by everyone (Arnao and Hernandez-Ruiz, 2019). In order to explore whether melatonin is involved in regulating other plant hormones in the process of improving cotton tolerance to salt stress, we analyzed the expression of genes related to the hormone pathway. We have observed that 11 hormone-related genes are differentially expressed by melatonin under salt stress (Figure 4), and five genes have decreased expression under salt stress, but are induced by melatonin to undergo differential changes under salt stress. Four genes (GH_A03G2091.gene, GH_A07G0419.gene, GH_D08G2786. gene, and GH_d01G1143. gene) were induced by melatonin to up-regulate expression, and 1 gene (GH_A08G2800.gene) was down-regulated and increased by melatonin. Six genes were not differentially expressed under salt stress, but they were induced by melatonin under salt stress. Four genes (GH_A13G0422.gene, GH_D04G1779.gene, GH_D05G2097.gene, and GH_D13G0417.gene) were up-regulated, and two genes (GH_A09G2113.gene and GH_A09G1643.gene) down-regulate the expression. These genes were involved in the signal transduction process of auxin, ABA, ethylene, brassinosteroid, and jasmonic acid.

We also found that in the brassinolide biosynthetic pathway, three genes were induced by melatonin, one gene (GH_A10G2603.gene) was down-regulated, and two genes (GH_A05G1364.gene and GH_D05G1368.gene) were up-regulated. At the same time, there were four genes (GH_A13G0422.gene, GH_D04G1779.gene, GH_D05G2097.gene, and GH_D13G0417.gene) in the signal transduction pathway of brassinolide under salt stress induced by melatonin to up-regulate their expression, but they were not induced by salt stress. Based on these results, we speculated that melatonin may act synergistically with brassinosteroid to improve cotton’s tolerance to salt stress.

### Melatonin-Regulated Genes Involved in Phosphoinositide Metabolism and Its Signaling System

**FIGURE 5:**
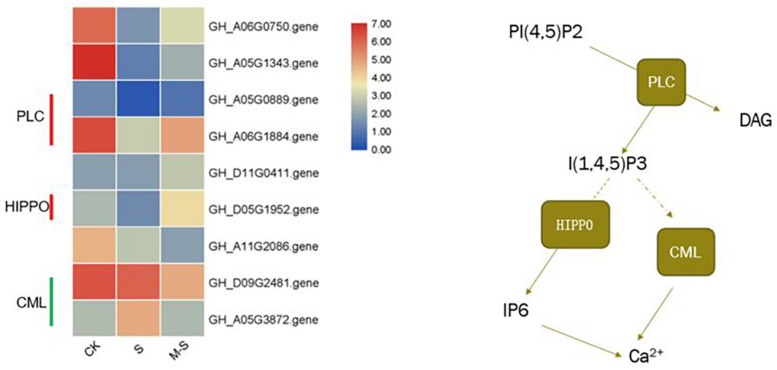
Effect of melatonin on the expression of phosphatidylinositol Signaling system related genes.

In order to explore which molecular mechanism of melatonin regulating to improve the salt tolerance of cotton, we enriched the differential genes between S and M-S into KEGG pathways. We found that seven genes (six genes up-regulated and one gene down-regulated) were enriched in Inositol phosphate metabolism (ko00562), and five genes (three genes up-regulated, two genes down-regulated) were enriched in phosphatidylinositol signaling system (ko04070). There were three genes that are commonly enriched in these two pathways (GH_A05G0889.gene, GH_A06G1884.gene, and GH_D05G1952.gene), all of which were up-regulated by melatonin under salt stress (Figure 5). Figure 5 showed the expression pattern of nine genes induced by melatonin in the process of phosphatidylinositol signaling system in cotton resisting salt stress. These genes were specifically expressed by melatonin under salt stress, indicating that melatonin participates in the resistance of cotton to salt stress by inducing phosphoinositide metabolism and the expression of genes related to signaling system.

### Detection of Endogenous Melatonin Content in Upland Cotton

**FIGURE 6:**
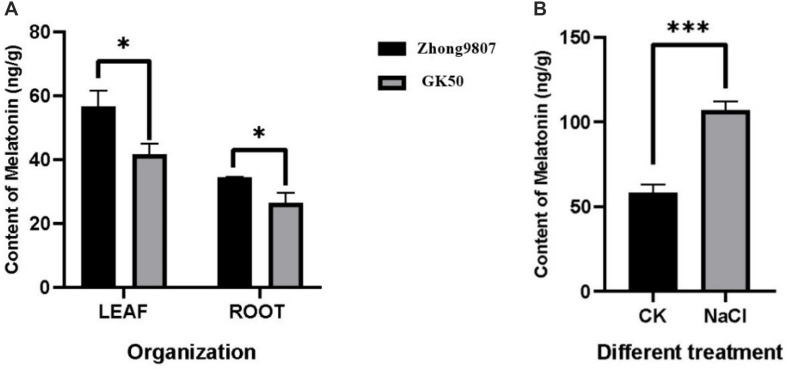
Determination of endogenous melatonin content in cotton. **(A)** Comparison of endogenous melatonin levels between two cotton species (Zhong9807 and GK50). **(B)** Effects of salt stress on endogenous melatonin levels in cotton seedlings (Zhong9807). *p < 0.05 and ***p < 0.001.

In order to explore whether the endogenous melatonin content is related to the salt tolerance of cotton, the endogenous melatonin levels of two cotton variety (Zhong9807, GK50) at the three-leaf stage seedlings were measured (Figure 6A). By comparing two cotton varieties with different salt tolerance, the melatonin content in Zhong9807 roots and leaves of the better salt tolerance is higher than GK50. We speculated that the salt tolerance of cotton may be related to the endogenous melatonin content. At the same time, in order to explore whether the endogenous melatonin content of cotton is involved in the resistance of salt stress, we treated the cotton seedlings at the three-leaf stage with salt, and measured the endogenous melatonin content after 12 h, and treated the water as a control. The results showed that salt treatment induced an increase in endogenous melatonin content (Figure 6). In short, cotton endogenous melatonin participates in cotton’s resistance to salt stress and has a certain relationship with cotton’s salt tolerance.

### Inhibition of Melatonin Made Cotton More Sensitive to Salt Stress

**FIGURE 7:**
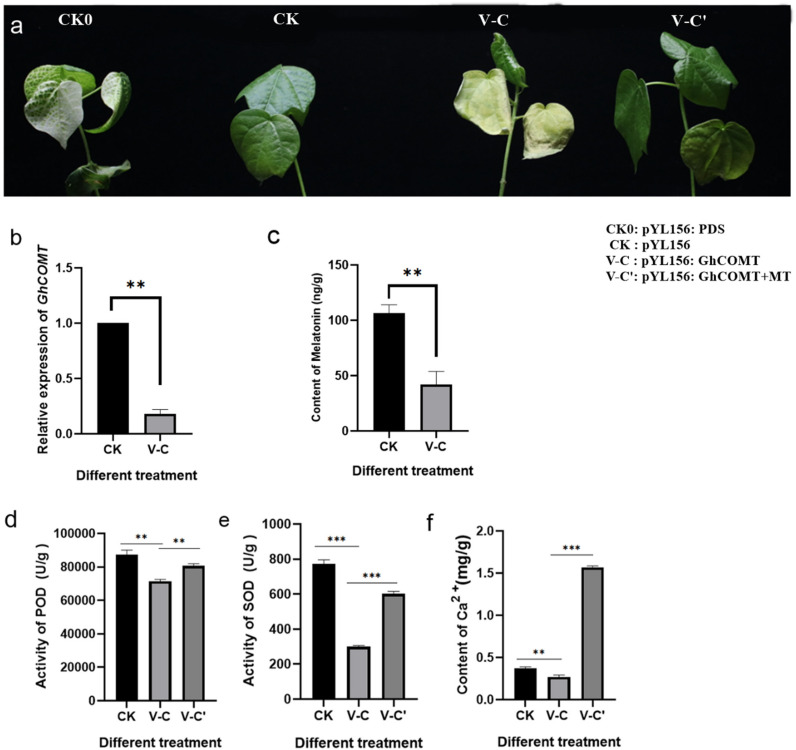
Inhibition of endogenous melatonin reduced the salt tolerance of cotton. **(a)** Salt tolerance of *GhCOMT* silenced plants decreased; **(b)** Detection of *GhCOMT* silencing efficiency; **(c)** Detection of melatonin content in *GhCOMT* silenced plants; **(d)** Detection of POD activity in *GhCOMT* silenced plants; **(e)** Detection of SOD activity in *GhCOMT* silenced plants; **(f)** Detection of melatonin content in *GhCOMT* silenced plants. **p < 0.01 and ***p < 0.001.

In order to investigate whether the change of endogenous melatonin content would affect the salt tolerance of cotton, VIGS technique was used to silence the melatonin synthesis gene *GhCOMT:pYL156:PDS* causes chlorosis and blanching of leaves, and the success of the experiment could be judged by observing whether the leaves of *pYL156:PDS* transformed plants turned white, with pYL156 empty injected plants serving as a blank control. About 2 weeks after VIGS infection, the leaves of *PYL156:PDS* plants became albino, indicating that our silencing system was stable. When the cotton seedlings grew to the three-leaf stage, the expression of *GhCOMT* was detected, and PYL156 plants were taken as the control, and it was found that the expression of *GhCOMT* was significantly decreased (Figure 7a). Meanwhile, the content of endogenous melatonin was significantly decreased in the silenced plants (Figure 7b), indicating that gene silencing was successful. Subsequently, the silencing plants were selected and subjected to salt stress, and the pYL156 and *pYL156:GhCOMT* plants were washed and transplanted into 300 mL conical flask containing 100 mM/L NaCl solution. At the same time, some of the *pYL156:GhCOMT* plants were exogenous with 20 μM melatonin, and the phenotype was obvious after 3 days of salt treatment (Figure 7c). The stress of gene silencing *pYL156:GhCOMT* plants was more serious than that of control pYL156 plants, and the degree of true leaf wilting was significantly higher than that of control plants. Meanwhile, the stress symptoms of *pYL156:GhCOMT* plants were alleviated after exogenous melatonin was applied.

Based on our transcriptome data, we found that melatonin enhances cotton salt tolerance mainly by affecting reactive oxygen scavenging system and Ca^2+^ signal transduction pathway. We measured biochemical markers POD activity (Figure 7d), SOD activity (Figure 7e), and Ca^2+^ content (Figure 7f) by sampling plants treated with pYL156, *pYL156:GhCOMT*, and *pYL156:GhCOMT*+20 μM melatonin, respectively. The POD activity, SOD activity and Ca^2+^ content of *pYL156:GhCOMT* plants were significantly down-regulated, indicating that the function of reactive oxygen scavenging system was weakened, Ca^2+^ signal translocation was weakened, and the salt tolerance of cotton was decreased. Meanwhile, exogenous melatonin supplementation could alleviate the effect of gene silencing on salt tolerance of cotton.

## Discussion

### Melatonin Plays an Important Role in Salt Tolerance of Cotton

Since melatonin is involved in many plant development processes and stress responses, melatonin levels in plants will change under different environments, especially under stress. It has been reported that sodium chloride, hydrogen peroxide, drought, pH, cold stress and ultraviolet radiation can all induce the increase of melatonin level in plants (Arnao and Hernández-Ruiz, 2006; Arnao and Hernández-Ruiz, 2013; Riga et al., 2014). Under various stress conditions, the level of melatonin in plants increases significantly (Zhang et al., 2015), which is conducive to anti-stress ability. This is consistent with our results, we found that salt stress can induce the increase of endogenous melatonin levels in cotton. At the same time, the content of endogenous melatonin in Zhong9807 with higher salt tolerance was higher than that in GK50, which also indicated that the content of melatonin in cotton with higher salt tolerance was higher, which was consistent with the conclusion that melatonin levels in *Verticillium dahliae* resistant varieties were higher than those in susceptible varieties (Li et al., 2019). Overexpression of melatonin synthesizing gene Caffeinate O-methyltransferase 1 (*COMT1*) significantly enhanced the ability of tomato to reduce the toxicity and residue of MBC (Yan et al., 2019). After drought treatment, the Pro content of transgenic *TACOMT* was higher than that of WT, while the MDA content was lower than that of WT (Yang et al., 2019). By silencing *GhCOMT*, we reduced the melatonin content of cotton, thus making silenced plants more sensitive to salt stress. These results fully show that melatonin is closely related to salt tolerance of cotton.

### Melatonin Acts as an Antioxidant Defense System in Cotton Salt Stress

Plant melatonin is considered to be an antioxidant defense system, which plays an important role in controlling ROS and reactive nitrogen species (RNS) in plant cells, as well as other free radicals and harmful oxidative molecules (Reiter et al., 2014). Studies have found that melatonin may reduce the oxidative damage caused by salt stress by directly enhancing the activity of antioxidant enzymes or removing H_2_O_2_ (Li et al., 2012). Exogenously apply of melatonin, regulates SOD, CAT, GR, APX, and ASC-GSH pathways, participates in enhancing the detoxification effect of ROS, reducing cell damage and cell death (Siddiqui et al., 2019). Melatonin pre-treatment of apple seedlings under salt stress are in high branch height, leaf number, chlorophyll content and electrolyte permeability affected by salt stress is less than the untreated plant, hydrogen peroxide level by half, to induce ROS metabolism enzyme (ascorbic acid peroxidase, catalase and peroxidase activity), Na^+^ and K^+^ transporter (NHX1 and AKT1) increases, it all helps to alleviate salinity induced inhibition (Li et al., 2012). The overexpression of *MzASMT9* led to the increase of melatonin, as a result, *Arabidopsis thaliana* overexpressed plant showed higher salt tolerance, lower ROS, lower lipid peroxidation and enhanced photosynthesis than wild-type plants (Zheng et al., 2017).

In this study, melatonin regulated the expression of redox-related genes and up-regulates genes related to the glutathione metabolism (ko00480) pathway to help cotton alleviate salt stress. Based on RNA-seq data, exogenous application of melatonin can change the expression of some genes related to redox and Glutathione metabolism, and even induce the expression of some genes that were not induced by salt stress, and participate in redox reactions. When plants are exposed to abiotic or biotic stress or sense melatonin, melatonin can regulate and rapidly up-regulate the activities of different antioxidant enzymes and stress tolerance related genes, and activate downstream signal transduction pathways (Arnao and Hernández-Ruiz, 2015). Due to its unique redox and nucleophilic properties, glutathione plays an important defensive role against ROS, foreign organisms and heavy metals in biological reduction reactions (Gao et al., 2020). In short, as an antioxidant defense system, melatonin improved the salt tolerance of cotton by scavenging active oxygen.

### Melatonin Interacts With Plant Hormones

With the melatonin receptor CAND2/PMTR1 discovered in *Arabidopsis* (Wei et al., 2018), Melatonin began to be considered a plant hormone. The structural similarity between melatonin and indole-3-acetic acid (IAA) (auxin) has prompted botanists to further study its possible role as a regulator of normal plant growth and development (Sun et al., 2020). Some studies have shown that plant melatonin works by regulating various elements related to the redox network or interfering with the activity of other plant hormones (Tan and Reiter, 2020). Melatonin maintains abscisic acid homeostasis by positively regulating its biosynthetic genes and negatively regulating catabolism genes. It effectively down-regulates the abscisic acid synthesis gene MdNCED3 and up-regulates its catabolism genes MdCYP707A2 and MdCYP707A1, resulting in a decrease in abscisic acid (Li et al., 2015). Drought stress up-regulates ABA, BRs, and JA, and down-regulates CKs and GAs, while melatonin increases BRs, GAs, JA, and CKs levels and reduces ABA levels (Moustafa-Farag et al., 2020). The melatonin-jasmonic acid interaction is expressed by regulating molecular transcripts such as JAZs in the jasmonic acid signal (Shi et al., 2015). Drought stress inhibits the biosynthesis of gibberellin, and after melatonin treatment, the biosynthesis of gibberellin is greatly enhanced, thereby improving drought tolerance (Sharma et al., 2020). Application of melatonin significantly increased the zeatin + zeatin riboside (Z + ZR), IAA, gibberellic acid (GA) contents (Ahmad et al., 2020). Melatonin is a positive regulator of dark growth or shade outgrowth by regulating BR biosynthesis in plants (Hwang and Back, 2018). The effect of melatonin on ethylene biosynthesis, ethylene perception and ethylene signal may help tomato fruit ripening and quality improvement (Liu et al., 2019).

Based on transcriptome data analysis, under salt stress, melatonin can induce the synthesis of some hormones (Auxin, ABA, Ethylene, Brassinosteroid, Jasmonic acid) and the expression of signal transduction-related genes. Some genes were not induced by salt stress, but their expression was induced by melatonin under salt stress. We speculated that melatonin may interact with other hormones by regulating the expression of these genes. Exogenous application of melatonin up-regulated the expression of cotton brassinolide synthesis-related genes and their signal transduction-related genes. In our study, two genes in the brassinolide synthesis pathway were up-regulated and expressed by melatonin, and one gene was down-regulated. At the same time, four genes in the brassinolide signal transduction pathway were up-regulated. Exogenous melatonin treatment induces multiple BRs biosynthetic genes, including DWARF4, D11, and RAVL1 (Hwang and Back, 2018). Melatonin regulated brassinolide synthesis, controls stomatal movement, improves cell membrane stability and water absorption, and reduces ion leakage caused by cell membrane damage under water-limited conditions (Moustafa-Farag et al., 2020). BRs can modify enzymatic and non-enzymatic antioxidant systems, which are involved in maintaining ROS homeostasis and protecting cells from ROS-induced damage (Núñez et al., 2003). As a hormone, melatonin is a regulator of many plant hormones (Arnao and Hernández-Ruiz, 2020), co-ordinating other plant hormones in response to salt stress in regulating cotton defense network.

### Melatonin as a Regulator Regulates Transcription Factors Involved in Salt Stress

Melatonin appears to be more than just a classic plant hormone, because its role is diverse, its potential to alter gene expression is significant, acting as a biological stimulus in non-biological stress situations, regulating key gene expression against stressors (Arnao and Hernandez-Ruiz, 2019). Transcriptome profile through RNA-sequence analysis identified 1228, 1120, and 1537 DEGs in control plant (Ctr) vs. simulated acid rain stressed plant (P25) comparison, control plant vs. melatonin treatment in simulated acid rain stressed plant (P25M) comparison and P25 vs. P25M comparison, respectively (Debnath et al., 2020). Transcription factors play an important role in stress tolerance. The reported transcriptome analysis confirmed that more than 30 TF family genes are involved in abiotic stress responses, including MYB, WRKY, ERF, bZIP, etc. (Peng et al., 2014). Many transcription factors have been confirmed to be up-regulated in melatonin therapy, most of which are stress-related transcription factors. Exogenous melatonin can regulate the expression of TFs such as bZIP, MYB, WRKY, ERF, promote the expression of genes encoding ROS scavenging enzymes, and improve tolerance to abiotic stress (Liang et al., 2015).

In this study, we found that 21 transcription factor families between S and M-S, with a total of 98 genes differentially expressed, of which 60 transcription factors were not induced by salt stress, but were specifically induced by melatonin under salt stress expression, such as AP2/ERF-ERF, C2H2, bHLH, and WRKY and other transcription factors. It is known that AP2/ERF family transcription factors regulate various environmental stress response processes of higher plants, such as abiotic stress (cold, heat, drought, salt, and osmotic stress) and biotic stress (herbivorous insects and microbial pathogens) (Feng et al., 2020). Studies have shown that C2H2 plays an important role in the defense and adaptation responses of plants to various environmental stress conditions (Wang et al., 2019). WRKY transcription factors have multiple functions in regulating stress response, leaf senescence, plant growth and development, etc. (Gu et al., 2018). Some bHLH TFs are also believed to be able to cope with a variety of abiotic stresses and improve the drought resistance, salt tolerance and cold tolerance of plants (Sun et al., 2015). These transcription factors induced by melatonin may play an important role in the resistance of cotton to salt stress.

### Melatonin as a Signaling Molecule Induces a Second Messenger in Response to Salt Stress

Many lipids and lipid-related molecules are thought to play a role in plant defense signals (Shah, 2005). Membrane-associated phospholipids and soluble inositol phosphates (collectively referred to as phosphoribosyls) are present in all eukaryotic cells and are involved in the response of plants to many environmental stimuli. When plants are under abiotic stress, membrane receptors will be stimulated, and membrane-related inositol phospholipids will transmit cellular information by producing second messengers, lipid-binding DAG and soluble IP3 (Jia et al., 2019). Phospholipase C (PLC) is activated by external stimuli to cause hydrolysis of PtdInsP2, generating soluble second messengers inositol 1,4,5-triphosphate (InsP3) and diacylglycerol (DAG) (Hung et al., 2014). In plants, DAG is converted to phosphatidic acid (PA), which is an important signal molecule in plant abiotic and biotic stress (Testerink and Munnik, 2011), InsP3 may be further phosphorylated to form inositol hexaphosphate (InsP6), both InsP3 and InsP6 are thought to release Ca^2+^ from intracellular storage (Valentová and Martinec, 2007; Yang et al., 2010). When plants are exposed to external stimuli, they increase the intracellular Ca^2+^ concentration, which in turn activates a series of calcium-binding proteins, including Ca^2+^ sensors/decoders, protein kinases and transcription factors (Srivastava et al., 2013). Melatonin can change the expression of genes involved in signal transduction. Six stress receptors and 14 genes involved in calcium-dependent signaling are up-regulated by melatonin (Weeda et al., 2014). The interaction between melatonin and Ca^2+^ calmodulin has been shown to regulate many calcium-dependent cellular functions in animal cells (Krystyna et al., 2009).

In this study, transcriptome data analysis found that *GhPLC2* (GH_A05G0889.gene and GH_A06G1884.gene) and *GhHIPP02* (GH_D05G1952.gene) were down-regulated under salt treatment, but were up-regulated by melatonin under salt treatment. Phospholipase C (PLC) can hydrolyze phosphatidylinositol 4,5-bisphosphate [PtdIns(4,5)P2] into two second messengers, inositol-1,4,5-triphosphate (InsP3) and Diacylglycerol (DAG) (Munnik and Vermeer, 2010). *GhHIPP02* can catalyze the production of Inositol hexaphosphate (IP6), which can be used as a second messenger that affects the release of Ca^2+^ in cells, and is also the main storage form of phosphate in plant cells. At the same time, the unique role of InsP6 in plants is related to the potential of regulating plant hormone receptors. It can bind to F-box protein, participate in the reception of auxin, and play a role in auxin signal transduction (Tan et al., 2007). Both *GhCML10* (GH_D09G2481.gene) and *GhCML45* (GH_A05G3872.gene) were down-regulated by melatonin under salt stress. Studies have shown that transgenic lines overexpressing *CML10* show late flowering and reduce seed yield. Compared with WT, the ability to resist osmotic stress is reduced, indicating that *CML10* has a negative effect on the growth and stress response of *Arabidopsis* (Divi et al., 2010). Cotton pre-treated with melatonin can accelerate the synthesis of InsP3 and InsP6 and promote the release of Ca^2+^ by up-regulating InsP3 and InsP6 synthesis-related genes under salt stress. At the same time, melatonin induced the down-regulation of *GhCML* gene under salt stress, leading to the release of Ca^2+^. As a signal molecule, melatonin promoted the release of Ca^2+^ by regulating the phosphatidylinositol signal system and improves the salt tolerance of cotton.

### Melatonin Improves Cotton Salt Tolerance Through a Complex Network

Soil salinization is an increasingly serious global problem, because salinization hinders plant growth and development and reduces crop yields. Soil salt produces osmotic stress and toxic stress to plants, leading to plant growth inhibition, developmental changes, metabolic adaptation, and ion isolation or rejection (Munns and Tester, 2008). The effects of salt stress on plants mainly include osmotic stress, specific ion toxicity, nutritional imbalance and ROS (Abbasi et al., 2016). Exposure of plants to salt stress can cause excessive production of ROS, resulting in damage to plant cell membranes (Mittova et al., 2009). A large number of studies have revealed the important role melatonin plays in improving the salt tolerance of plants.

**FIGURE 8:**
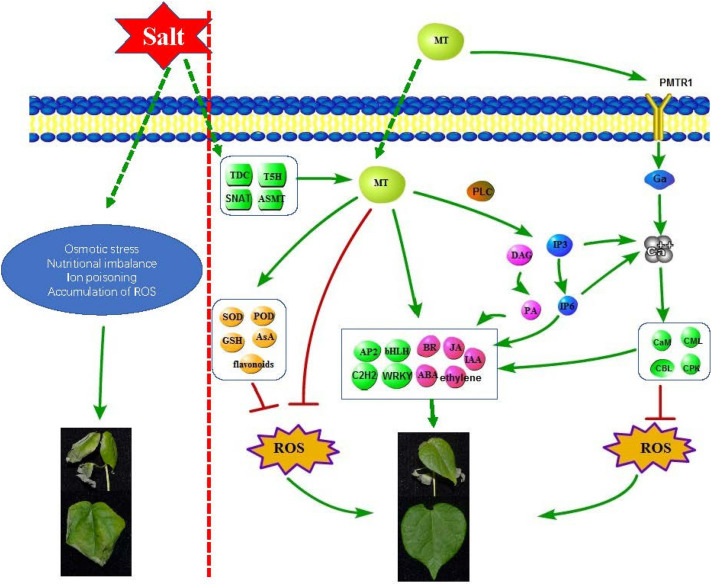
Schematic diagram of molecular mechanism of melatonin enhancement of cotton salt tolerance.

In this study, combining our results, a complex regulatory network was used to describe the molecular role of melatonin in improving the salt tolerance of cotton (Figure 8). We found that salt treatment can induce an increase in the level of endogenous melatonin in cotton seedlings; exogenous application of melatonin can regulate antioxidant enzymes, redox-related genes, glutathione, etc. In order to remove ROS, melatonin can also induce the accumulation of some representative non-enzymatic antioxidants, such as AsA, phenolic compounds, flavonoids and carotenoids (Pardo-Hernández et al., 2020); as a broad-spectrum antioxidant, interacts with ROS and directly eliminates it (Lins et al., 2012); melatonin induced the expression of some hormones (Auxin, ABA, Ethylene, Brassinosteroid, Jasmonic acid) synthesis and signal transduction related genes, and coordinates other hormones in the cotton defense network; melatonin induced the expression of some transcription factors (AP2/ERF-ERF, C2H2, bHLH, WRKY, etc.) to resist salt stress; melatonin induced the expression of genes related to the phosphatidylinositol signaling system and activates some signaling molecules (IP3, DAG, IP6, Ca^2+^), Phospholipase C (PLC) hydrolyses PtdInsP2 to generate InsP3 and DAG, which can be converted into PA as a signal molecule and PA as a membrane localization signal. Many PA target proteins (AtPDK1, ABI1, CTR1, etc.) involved in environmental stress response have been identified (Hou et al., 2016), and InsP3 generates InsP6 through a series of enzymatic reactions. InsP6 is the main storage form of phosphate in plants and the second messenger of plants. It can regulate Ca^2+^ release, hormone receptor TIR1, gene expression, etc. (Munnik and Vermeer, 2010). When melatonin is sensed by receptor CAND2/PMTR1, it triggers the dissociation of Gα form Gγβ, which activates the downstream H_2_O_2_ and Ca^2+^ signaling transduction cascade, leading to the phenotype of stomatal closure (Wei et al., 2018). Intracellular Ca^2+^ increases, thereby initiating stimulation-specific downstream signal transduction. This is dependent upon the array of calcium sensors. The calcium sensors are categorized into CaMs (calmodulins), CMLs (CaM-like proteins), CDPKs (Ca^2+^ dependent protein kinases), and CBLs (calcineurin B-like proteins) (Srivastava et al., 2013). These sensor proteins can directly perform their functions or interact with corresponding decoding elements. The application of melatonin and Ca^2+^ inhibits ROS generating enzymes, including NADPH oxidase in the plasma membrane and GOX in peroxisomes, respectively (Siddiqui et al., 2020). In this study, we found that melatonin melanin plays an important role in improving the salt tolerance of cotton, laying a certain theoretical foundation for improving the salt tolerance of cotton.

## Conclusion

As an antioxidant defense system and signaling molecule, melatonin plays an indispensable role in the response of plants to abiotic stress, and has great potential in improving crop response to abiotic stress. The content of endogenous melatonin in cotton may affect its salt tolerance. In this study, we used exogenous application of melatonin to increase the salt tolerance of cotton, and inhibiting the content of endogenous melatonin decreased the cotton salt tolerance, indicating that melatonin and cotton salt tolerance are closely related. RNA-seq technology was used to explore the mechanism of melatonin regulating the salt stress of cotton at the molecular level. This study found that melatonin improved cotton’s tolerance to salt stress by regulating antioxidant systems, plant hormones, transcription factors, lipid metabolism, signal molecules and other mechanisms. This study is helpful to establish an effective pathway to improve cotton salt-tolerance through melatonin mediation.

## Data Availability Statement

The datasets presented in this study can be found in online repositories. The names of the repository/repositories and accession number(s) can be found below: NCBI SRA BioProject, accession no: PRJNA722118.

## Author Contributions

WY and YZ: conceived and designed the experiments. YZ, XL, XC, and JW: methodology. YZ, YF, CR, HZ, and NX: experiments. MD, DW, JW: analysis of data. YZ: writing—original draft preparation. YZ, QW, SW, CC, LG, and LZ: writing—review and editing. WY: supervision. All authors contributed to the article and approved the submitted version.

## Conflict of Interest

The authors declare that the research was conducted in the absence of any commercial or financial relationships that could be construed as a potential conflict of interest.

## Footnotes

1 http://www.genome.jp/kegg/

2 https://vigs.solgenomics.net/
