# Supplementary material for: Melatonin Improves Cotton Salt Tolerance by Regulating ROS Scavenging System and Ca2 + Signal Transduction
Source: Front Plant Sci. 2021 Jun 28;12:693690. doi: 10.3389/fpls.2021.693690 (PMC8273866; doi:10.3389/fpls.2021.693690)
Supplement: Supplementary file 1 [file Data_Sheet_1.docx]

Supplementary Material

# Supplementary Data

Supplementary Table 1 Summary of RNA-seq data

| Samples | Clean reads | Clean bases | GC Content | %≥Q30 |
| --- | --- | --- | --- | --- |
| CK1 | 21,609,548 | 6,471,301,376 | 44.68% | 93.11% |
| CK2 | 19,576,573\ | 5,862,497,894 | 44.77% | 93.06% |
| CK3 | 19,475,405 | 5,832,800,220 | 44.40% | 93.04% |
| S1 | 21,721,981 | 6,505,956,250 | 44.62% | 93.11% |
| S2 | 22,157,942 | 6,602,027,482 | 44.34% | 94.83% |
| S3 | 20,508,902 | 6,142,285,234 | 44.97% | 93.21% |
| M-S1 | 24,237,490 | 7,260,756,872 | 44.56% | 93.76% |
| M-S2 | 21,591,069 | 6,451,768,316 | 44.70% | 94.73% |
| M-S3 | 26,212,950 | 7,851,413,718 | 44.49% | 93.03% |

Supplementary Table **2 Primer sequence of qRT-PCR**

| Gene ID | Forward primer (5’- 3’) | Reverse primer (5’- 3’) |
| --- | --- | --- |
| GH_A12G1390 | GGAGACGAAGACGAAGACGA | GTCGCCGTTGTATGGTGTTG |
| GH_D02G2249 | CGATTCGGAAACCCTGTTCC | CGGTTTCATCGCTCAACTGT |
| GH_A09G2251 | GGGAGCGTTGATATGTCTGC | CCCGACATACGATTGACAGC |
| GH_A12G1390 | GGAGACGAAGACGAAGACGA | GTCGCCGTTGTATGGTGTTG |
| GH_A01G0104 | CGGCAGCAACAGTTATGGTT | CACTACCACCGCTAGGTGAA |
| GH_D11G0933 | GCCCTTTGACACACTCATCC | CTTGATTAGCCGCTGCAGTT |
| GH_D13G0150 | CCACTGTTGCAACGACTTCA | ACAACGATCCCACCAATCCT |
| GH_A10G2593 | GCGAGGCCTTGAAGAACATT | AGGTTTCTCACCGTCTCTGG |
| GH_D03G1742 | ATTAGACGAGCAGCTGTGGA | CTAGCAAGCAAGTTCGGCAT |
| GH_A06G0862 | AAGCGATTCACGGTTCAAGG | CGGGAGGGTTATCAGGGAAG |
| GH_D05G1368 | ACTCCATTTGGTGGTGGACA | CCCAAACCGGCATTCTCTTC |
| GH_D05G3643 | TTGGCGGTCCTCAAATTGTC | GCCACAAGAGACACCAATCC |
| GH_D13G0417 | AACAATGGCGGAAACCCTTC | TGCTAGGGACTCATCGGTTC |
| GH_A10G2606 | CTCGCGAACCGAAACTTGTA | GTGGCAATCCTTTGGTGGTT |
| GH_D11G2757 | CAGCTGGCATTGGAATCCTC | GTTTACGATCCGCTGTGCTT |
| GhUBQ7 | CGCTGTACTTCTACTCCC | GAATGCCTTCCTTGTCTT |

Supplementary Table **3 Expression of melatonin-regulated REDOX genes under salt stress**

| Gene ID | CK (FPKM) | NaCl (FPKM) | MT+ NaCl (FPKM) |
| --- | --- | --- | --- |
| GH_D08G0545.gene | 0.39 | 0.05 | 0.78 |
| GH_D12G0582.gene | 1.06 | 0.31 | 1.49 |
| GH_A05G1364.gene | 0.06 | 0.32 | 1.14 |
| GH_D02G0200.gene | 2.57 | 0.09 | 1.98 |
| GH_A08G0541.gene | 0.38 | 0.09 | 0.75 |
| GH_D03G0079.gene | 1.08 | 0.70 | 3.37 |
| GH_A13G0290.gene | 1.71 | 0.43 | 2.38 |
| GH_D10G2246.gene | 22.55 | 1.21 | 9.74 |
| GH_A06G0750.gene | 58.52 | 2.13 | 8.35 |
| GH_A05G1343.gene | 123.59 | 1.25 | 3.78 |
| GH_A11G0486.gene | 104.50 | 44.35 | 14.08 |
| GH_A02G0401.gene | 14.20 | 0.06 | 4.19 |
| GH_D02G0353.gene | 2.44 | 0.93 | 2.38 |
| GH_D05G1622.gene | 158.30 | 29.48 | 79.90 |
| GH_A12G0340.gene | 6.06 | 1.39 | 0.22 |
| GH_A07G0935.gene | 121.81 | 15.26 | 5.76 |
| GH_D12G0372.gene | 12.81 | 2.66 | 0.28 |
| GH_A06G1828.gene | 4.89 | 2.16 | 5.85 |
| GH_D05G1382.gene | 3.76 | 0.83 | 2.55 |
| GH_A11G0010.gene | 8.30 | 2.66 | 9.11 |
| GH_D04G0747.gene | 5.18 | 1.34 | 3.81 |
| GH_D13G0287.gene | 2.04 | 0.47 | 2.89 |
| GH_D09G2403.gene | 14.86 | 4.17 | 9.96 |
| GH_D09G1074.gene | 60.86 | 2.37 | 0.50 |
| GH_D10G0148.gene | 4.85 | 0.64 | 2.56 |
| GH_A05G1594.gene | 83.92 | 23.05 | 76.90 |
| GH_A09G1832.gene | 407.32 | 5.19 | 14.20 |
| GH_D09G1786.gene | 191.72 | 6.24 | 17.37 |
| GH_D04G1896.gene | 29.94 | 5.79 | 2.30 |
| GH_A08G0535.gene | 1.04 | 0.31 | 1.96 |
| GH_A06G1717.gene | 9.07 | 3.58 | 15.36 |
| GH_D05G1368.gene | 1.01 | 2.37 | 5.90 |
| GH_D03G0448.gene | 66.09 | 39.88 | 17.71 |
| GH_A12G0204.gene | 59.80 | 68.10 | 32.57 |
| GH_D08G2493.gene | 4.75 | 14.42 | 6.70 |
| GH_A04G1555.gene | 2.33 | 2.34 | 7.58 |
| GH_A10G2296.gene | 3.48 | 3.61 | 0.95 |
| GH_A07G2230.gene | 0.56 | 0.25 | 1.02 |
| GH_D12G2239.gene | 1.62 | 14.84 | 3.94 |
| GH_A05G2328.gene | 3.41 | 4.35 | 1.73 |
| GH_D11G1903.gene | 1.54 | 0.80 | 2.82 |
| GH_A11G1527.gene | 9.71 | 7.06 | 2.64 |
| GH_A11G1383.gene | 9.54 | 7.04 | 2.09 |
| GH_D09G1370.gene | 0.47 | 2.23 | 0.41 |
| GH_D11G2399.gene | 0.94 | 4.08 | 1.65 |
| GH_A04G1552.gene | 0.58 | 5.33 | 18.35 |
| GH_A05G1490.gene | 0.10 | 0.96 | 0.31 |
| GH_A02G0696.gene | 0.78 | 2.36 | 0.58 |
| GH_A10G2603.gene | 21.81 | 83.24 | 29.49 |
| GH_A01G0071.gene | 4.38 | 11.59 | 4.74 |
